# P-1219. Benchmarks for Humanized Meropenem Plasma and Pulmonary Epithelial Lining Fluid (ELF) Exposures Against *Klebsiella pneumoniae* in Standardized Murine Lung Infection Model

**DOI:** 10.1093/ofid/ofae631.1401

**Published:** 2025-01-29

**Authors:** Andrew J Fratoni, Alissa Padgett, Erin Duffy, David P Nicolau

**Affiliations:** Hartford Hospital, Hartford, Connecticut; Hartford Hospital, Hartford, Connecticut; CARB-X, Boston, Massachusetts; Hartford Hospital, Hartford, Connecticut

## Abstract

**Background:**

Inter-laboratory method variability in conducting preclinical murine infection models make data reproducibility and comparisons problematic. The collaboration for prevention and treatment of MDR bacterial infections (COMBINE) consortium have proposed a standardized global protocol to align fundamental elements across laboratories. We developed human simulated regimens (HSRs) based on both plasma and ELF exposures for meropenem in the COMBINE murine neutropenic lung infection model and provide benchmark efficacy against *Klebsiella pneumoniae* isolates.

**Table 1.**
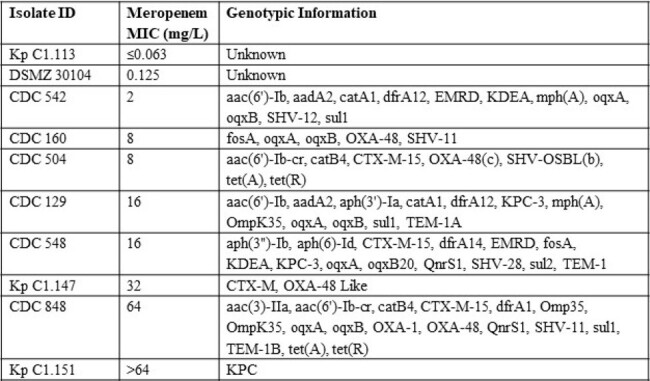
Phenotypic and genotypic information of Klebsiella pneumoniae isolates

**Methods:**

Following the COMBINE protocol for the preclinical murine lung model, neutropenic mice that received uranyl nitrate on day -3 to provide predictable renal impairment were administered 0.05 mL intranasal inoculums of *K. pneumoniae* (n=10) under isoflurane anesthesia. Isolate MICs were determined via BMD. Humanized exposures of meropenem 2g q8h as 3h infusion in plasma (65 mg/kg at 0 and 1.25h, 45 mg/kg at 3.5 and 6h, every 8h) and ELF (20 mg/kg at 0h, 50 mg/kg at 1.25h, 35 mg/kg at 3.5h, 20 mg/kg at 4.75h, and 15 mg/kg at 6h, every 8h) were administered to groups of 6 mice. Efficacy was measured in log_10_ CFU/lung at 24h compared with 0h controls. All isolates except CDC 160 and CDC 848 were tested in duplicate on separate days, with datasets combined and analyzed as one.

**Figure 1.**
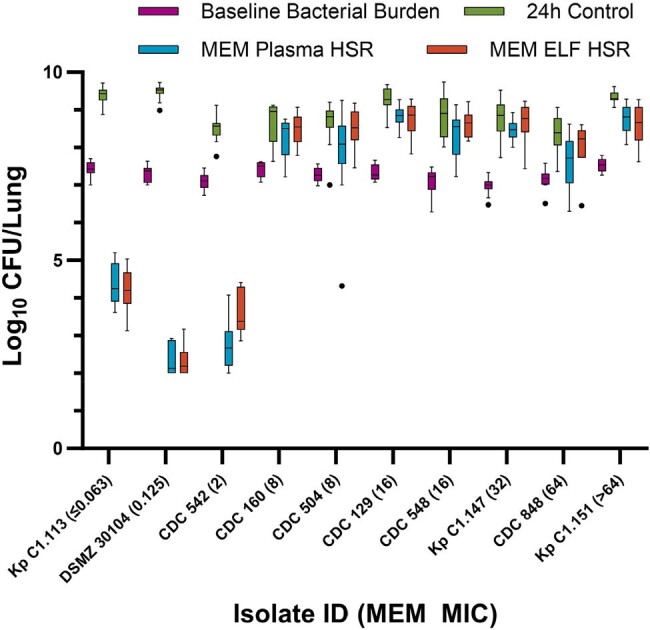
CFU/Lung data following administration of humanized meropenem (2g q8h 3h infusion) plasma and ELF exposures in the COMBINE murine neutropenic lung infection model. Outlier mice determined by Tukey’s Test and displayed as individual dots.

**Results:**

Meropenem MICs and genotypic information are presented in Table 1. Mean ± SD initial bacterial burden across all isolates were 7.27 ± 0.21 and 24h net growth in log_10_ CFU/lung in untreated controls was 1.72 ± 0.34. The CFU/Lung data for each isolate are presented in Figure 1. ≥ 1 log kill was achieved in all 3 non-resistant (MIC ≤ 2 mg/L) isolates and in none of the 7 resistant isolates. Numerical differences in CFU/Lung were seen between the meropenem plasma and ELF HSRs, however there were no categorical differences in achieving ≥ 1 log kill among these 10 isolates.

**Conclusion:**

Meropenem HSRs based on both plasma and ELF exposure were developed in the COMBINE neutropenic lung infection model and tested against a broad array of *K. pneumoniae* isolates. These data serve to provide a comparative benchmark for future small molecules in preclinical development against meropenem with these isolates and the standardized protocol.

**Disclosures:**

**Andrew J. Fratoni, PharmD**, InsightRX: Grant/Research Support **Erin Duffy, PhD**, CARB-X: Employee **David P. Nicolau, PharmD**, CARB-X: Grant/Research Support|Innoviva: Grant/Research Support|Innoviva: Honoraria|Merck: Advisor/Consultant|Merck: Grant/Research Support|Merck: Honoraria|Pfizer: Advisor/Consultant|Pfizer: Grant/Research Support|Pfizer: Honoraria|Shionogi: Advisor/Consultant|Shionogi: Grant/Research Support|Shionogi: Honoraria|Venatorx: Grant/Research Support

